# Sharing the Ride: *Ixodes scapularis* Symbionts and Their Interactions

**DOI:** 10.3389/fcimb.2020.00142

**Published:** 2020-04-08

**Authors:** Philip E. Stewart, Marshall E. Bloom

**Affiliations:** Biology of Vector-Borne Viruses Section, Laboratory of Virology, Rocky Mountain Laboratories, National Institute of Allergy and Infectious Diseases, National Institutes of Health, Hamilton, MT, United States

**Keywords:** *Babesia*, *Ehrlichia*, *Rickettsia*, deer tick virus, *Borrelia burgdorferi*, microbiome

## Abstract

The deer tick *Ixodes scapularis* transmits a variety of disease agents in the United States, spreading the bacteria that causes Lyme borreliosis, the protozoan agent of babesiosis, and viruses such as Powassan. However, a variety of other organisms have also evolved symbiotic relationships with this tick species, and it seems likely that some of these microbes have simultaneously coevolved mechanisms to impact each other and their tick host. The number of organisms identified as *I. scapularis* symbionts has increased seemingly exponentially with the advent of PCR and next generation sequencing technologies, but convincing arguments have proposed that some of these are of environmental origin, unadapted to surviving the physiological conditions of the tick or that they are artifacts of ultrasensitive detection methods. In this review, we examine the diversity of the known microbes occurring within the *I. scapularis* microbiome, the evidence for interactions between microbes, and discuss whether some organisms reported to be symbionts of *I. scapularis* are experimental artifacts.

## Introduction

The interactions of microbial symbionts with each other and with their hosts can be viewed analogously to passengers sharing a ride—they may or may not interact directly with each other; they may contribute to the cost or ride at the expense of another; they might help each other, ignore each other, or be antagonistic; they may sit together or segregate to different parts of the car; and they may get out at different destinations or stay in for the long haul. Symbiotic examples of these ride-sharing characteristics have been well-documented among arthropods and their microbiota. Specific instructive examples of beneficial interactions include two bacterial endosymbionts of the glassy-winged sharpshooter (*Homalodisca coagulata*) each of which synthesizes different essential metabolites for the host and possibly for each other (Wu et al., 2006) and a gut commensal bacterium of the *Aedes aegypti* mosquito that facilitates arboviral infection by altering the gut epithelial layer (Wu et al., 2019). An example of an antagonistic interaction in arthropods includes the inhibition of *Plasmodium falciparum* development within the midgut of *Anopheles gambiae* mosquitos resulting from generation of reactive oxygen species by an endogenous *Enterobacter* strain (Cirimotich et al., 2011).

While there has been intensive study of some arthropods' microbiota, these have typically focused on mosquitos (because of their role in pathogen transmission) or on agricultural pests, such as aphids. However, similar studies on tick microbiomes have only recently accelerated and still lag behind that of insects. *Ixodes scapularis*, commonly known as the black-legged or deer tick, is a major vector of human disease agents in the United States. In New York state, 22% of surveyed *I. scapularis* carried more than one human pathogen (Sanchez-Vicente et al., 2019). In some regions of North America, these polymicrobial infections occur at a higher prevalence than previously thought and may complicate both proper diagnoses and treatments. Identifying the full range of microorganisms that inhabit this tick species has practical implications for medical and veterinary health by helping to improve diagnosis, treatment and recovery. Characterizing the biological interactions between symbionts and the host tick may illuminate new strategies to prevent tick-borne diseases by inhibiting pathogen colonization of *I. scapularis* or for engineering paratransgenic organisms antagonistic to pathogens. Studies suggest that some microbes may be excluded from *I. scapularis* by the presence of others (Steiner et al., 2008; Narasimhan et al., 2014; Ross et al., 2018) and acquisition of a paratransgenic bacterium has been demonstrated for the hard tick *Hyalomma dromedarii* (Koosha et al., 2019).

A diversity of microbes is known to inhabit *I. scapularis*, either as a vehicle for transport or for permanent residence. This microbiome includes extracellular and intracellular bacteria, viruses, and eukaryotes. Bacteria infecting *I. scapularis* include the human pathogens *Borrelia burgdorferi* (agent of Lyme borreliosis) (Burgdorfer et al., 1982), *B. miyamotoi* (relapsing fever) (Scoles et al., 2001), and *Anaplasma phagocytophilum* (anaplasmosis) (Telford et al., 1996), while non-pathogenic, intracellular bacteria such as *Rickettsia buchneri* also inhabit this tick (Kurtti et al., 2015). The Deer tick virus (Powassan virus lineage II), a human viral pathogen, is also vectored by *I. scapularis* (Telford et al., 1997), as are viruses predicted to be symbionts (Tokarz et al., 2018). Eukaryotes identified within this tick species include protozoans, nematodes, and fungi. Here, we define “symbionts” as those organisms (mutualistic, commensal, parasitic, etc…) that require *I. scapularis* for their survival in nature. Similarly, we follow the definition of “microbiome” as set forth by Whipps et al. in 1988: “This may be defined as a characteristic microbial community occupying a reasonably well-defined habitat which has distinct physio-chemical properties. The term thus not only refers to the microorganisms involved but also encompasses their theater of activity” (Whipps et al., 1988). Therefore, we propose the microbiome of *I. scapularis* to be composed of both symbionts and free-living, environmentally acquired microbes that likely do not survive transstadially but persist while the bloodmeal nutrients are accessible. These relatively transient passengers may not be dependent on the tick for their survival in nature but may impact the tick or its symbionts and therefore may be viewed as potentially influential components of the microbiome. Some microbes are important human pathogens, but in this review we are focused on all the microbes and their relationships with the tick, though we do highlight human health impacts where relevant.

Less clear than the diversity of organisms, is the extent, if any, to which some of these microbes may interact with each other, either directly or indirectly. The published studies in this area are largely limited to predicted interactions based on co-occurrences of two or more organisms in the same tick (Cross et al., 2018; Ross et al., 2018). This review will focus on both the composition of the *I. scapularis* microbiome and the potential microbial interactions occurring within this tick species. Many of the organisms detected in microbiome studies are free-living environmental bacteria, and we discuss the reasons why some of these may artificially inflate the microbial diversity estimates. A further emphasis of this review is placed on interactions associated with *B. burgdorferi*, as this bacterium is perhaps the most studied symbiont of *I. scapularis* and an agent of Lyme borreliosis in the U.S. First, though, descriptions of the life cycle and the physiological environment that the internal anatomy of *I. scapularis* presents to its microbial inhabitants are both critical to understanding the composition of the microbiome and the interactions that may occur between its constituents.

## *I. scapularis* Life Cycle and Physiology

**Figure 1:**
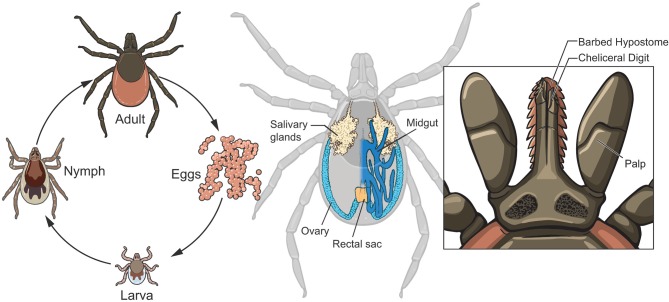
Life cycle and anatomical features of *Ixodes scapularis*. After hatching from eggs, *I. scapularis* progresses through 3 developmental stages: larval, nymphal and adult (left panel). Note the morphological differences that occur during each molt (see text for additional details): size successively increases and the larval-nymphal molt produces an additional pair of legs while sexual development occurs during the nymphal-adult molt. The midgut (dark blue with only the right half depicted, middle panel) is the site of digestion of the bloodmeal and is a significant entry route for microbes. The salivary glands (beige) are the exit ramps for transmission of infectious microbes to vertebrate hosts. Ovaries (light blue) are also organs of transmission for those symbionts transmitted to the progeny. The anatomy of the tick mouthparts (right panel) facilitates the feeding of *I. scapularis*, with the saw-like chelicerae cutting through host tissues and vessels to gain access to the blood.

After hatching from the egg, *I. scapularis* undergoes three developmental stages: larval, nymphal, and adult (Figure 1, left panel), with an average life span of ~2 years. A bloodmeal is required at each developmental stage to progress through the life cycle. The white-footed mouse, *Peromyscus leucopus*, is a primary host species for both the larvae and nymphs, while white-tailed deer (*Odocoileus virginianus*) are the maintenance host for adults (Piesman and Spielman, 1979; Piesman et al., 1979; Carey et al., 1980). However, *I. scapularis* feeds upon a variety of vertebrates including birds, lizards, and mammals. Once feeding is complete, the tick detaches and returns to the environment to digest the bloodmeal and molt to the next life stage. The molting process allows the tick to increase in size and produces acute physiological changes in the tick that must be tolerated by any symbionts. *I. scapularis* sexually develops during the nymphal molt, and the female requires a bloodmeal for fecundity although males do not need to feed. After mating the female deposits her egg mass (consisting of thousands of eggs) and dies, while the male may mate repeatedly.

Although blood feeding (hematophagy) is a characteristic of many arthropod species, ticks evolved a unique feeding style that merits description and has a significant impact on those microbiota that seek to infect the tick via ingestion of the blood into the midgut (Figure 1, middle) or that require the nutrients and signals from the blood. Lacking mandibles to bite with, *I. scapularis* instead use their saw-like chelicerae (Figure 1, right panel) to tear through the host skin and sever blood vessels, allowing the blood to pool into the bite site. While imbibing blood, the tick is also discharging saliva into the lesion, which has a profound effect on the host at the bite site as salivary components include immunomodulatory proteins and compounds that inhibit coagulation, pain and inflammation and may aid in the transmission of microbes from the tick to the host. The feeding process can last from several days to over a week, depending on the life stage, and this extended timeframe provides ample opportunity for microbes to be acquired by *I. scapularis*. Within the midgut lumen, the erythrocytes are lysed, but the remaining components are absorbed and digested intracellularly over an extended time period.

This mode of digestion makes the midgut lumen a relatively hospitable environment for microbes due to a neutral pH and the absence of digestive enzymes (Hajdusek et al., 2013), although hemoglobin moieties released during digestion of the bloodmeal have antimicrobial activity (Sonenshine et al., 2005). The brevity of tick feeding compared to its life span, and therefore infrequent access to nutrients, presents a limitation that organisms must overcome to establish a stable symbiotic relationship with *I. scapularis*. The peritrophic membrane is another limitation. Many arthropod species form a peritrophic membrane to separate the ingested blood from the epithelial cells of the midgut. This membrane may function to protect midgut cells from abrasive food particles and to limit dissemination of parasitic organisms that enter during feeding, preventing or reducing the number of microbes that escape to infect distal organs of the tick. Observed in all life stages of both *I. scapularis* and *Ixodes ricinus* (European species), initial formation of the peritrophic membrane is detected within 24 h after the onset of feeding (Rudzinska et al., 1982; Zhu et al., 1991; Sojka et al., 2007; Yang et al., 2014). Presumably, those microbes that seek to disperse throughout the tick body must do so either before the peritrophic membrane has fully solidified or after it breaks down. *Babesia microti*, the agent of human babesiosis and a symbiont of *I. scapularis*, is the only organism with the demonstrated capability to traverse the fully formed barrier (Rudzinska et al., 1982). Using a highly specialized organelle, termed the arrowhead, *B. microti* appears to cleave through the membrane and penetrate the epithelial lining of the midgut. Recent studies suggest that both *B. burgdorferi* and *A. phagocytophilum* may also penetrate the peritrophic membrane by unknown mechanisms (Abraham et al., 2017; Narasimhan et al., 2017).

Microbes that have evolved to establish residency within the tick must be tolerated by the host's innate immune defenses or have a mechanism to avoid or cope with it. The immune system of *I. scapularis* includes hemocyte cells (some of which are capable of phagocytosis), antimicrobial peptides and compounds, reactive oxygen and nitrogen species, lysozyme, peptidoglycan-recognition proteins, and small interfering RNAs (Johns et al., 2001; Schnettler et al., 2014; Bourret et al., 2016; Gulia-Nuss et al., 2016). The densities of some microorganisms are known to be controlled by factors of the immune system but still establish residency. *A. phagocytophilum*, an intracellular symbiont of *I. scapularis*, is limited by a member of the 5.3 kDa family of antimicrobial peptides expressed in the salivary glands (Liu et al., 2012). The extracellular spirochete *B. burgdorferi* can be phagocytosed by tick hemocytes (Johns et al., 2001), and is believed to be cleared by this mechanism from the hemocoel of the tick after feeding. However, *I. scapularis* hemocytes are less effective at killing *B. burgdorferi* than those of *Dermacenter variabilis* (which is not a competent vector for this bacterium), suggesting that the former tick may have evolved a level of immunotolerance to *B. burgdorferi*.

In addition to the barriers presented by the midgut and the innate immune system, feeding and molting produce substantial changes in *I. scapularis* anatomy. Passive oxygen diffusion from the surface of the tick is sufficient for the physiologic needs of the larval tick but occurs too slowly for the increased size of the nymph. Therefore, spiracles and the associated tracheal system (a branched, air-filled tubular network) develops during the larval molt and facilitates gas exchange to all tissues and organs. An additional pair of legs also develop during the larval molt. During the nymphal molt, gender development occurs. Shedding of the old cuticle and generation of a “new” exoskeleton occurs in every molt. The salivary gland acini (responsible for secretion during feeding) are a microbial transmission route from the tick and degenerate after feeding. These secretory acini reform before *I. scapularis* feeds again. From a microbial viewpoint, disintegration of the salivary gland acini means that permanent residency for symbionts seeking transmission from the tick cannot be maintained at this site but must be established elsewhere and reinfect these acini prior to or during feeding.

The successful transmission cycle of the spirochete *B. burgdorferi* is illustrative of the adaptations required for a symbiont to overcome the difficult and changing environment of *I. scapularis*. *B. burgdorferi* may be acquired by larval *I. scapularis* when it feeds on an infected vertebrate, and the bacterium will then establish residency in the midgut. Because *B. burgdorferi* lacks cellular biosynthetic pathways (Fraser et al., 1997), the bacterium appears to be dependent on the tick or the bloodmeal components to acquire certain nutrients and metabolic intermediates for survival. Using gas chromatography coupled to mass spectrometry, Hoxmeier et al. identified reductions in levels of compounds in the tick that correlate to the presence of *B. burgdorferi*, including purine metabolites, hydrophobic and aliphatic amino acids, galactose, glycerol and maltose, and specific fatty acids (Hoxmeier et al., 2017). With the depletion of the bloodmeal and the physiological changes that occur during the molt, *B. burgdorferi* densities decline (Piesman et al., 1990). To maintain required metabolic activities when the bloodmeal is depleted, *B. burgdorferi* may switch from using glucose as a carbon source to glycerol, which is present in the hemolymph (Pappas et al., 2011). When the nymphal tick begins to feed, the spirochete populations increase and *B. burgdorferi* evades entrapment by the peritrophic membrane and escapes the midgut, migrates into the hemocoel and from there penetrates the regenerated salivary glands for transmission to the vertebrate host. After the tick has completed its feeding, those *B. burgdorferi* that remain outside the midgut are eventually destroyed, likely by the hemocyte immune cells (Johns et al., 2001). However, the spirochetes remaining in the midgut act as a seed population during the next *I. scapularis* bloodmeal and can outgrow and again infect the salivary glands for potential transmission. Like *B. burgdorferi*, other microbes have adapted to these barriers present by *I. scapularis* physiology and established a symbiotic relationship with this tick species.

## Microbiota of *I. scapularis*

Although the composition and dynamics of the *I. scapularis* microbiome is still being defined, it is clearly diverse, with intra- and extra-cellular bacteria, viruses, fungi, nematodes, and protozoa having been identified by various methods (Table 1). We have included microbes that were detected in >1% of the ticks in each study and that have been corroborated by multiple studies or by more than one technique. However, we have excluded common environmental microbes demonstrated to be artifacts from contaminated reagents, such as *Delftia, Comamonas, Acidovorax*, and others (Salter et al., 2014). Sequence-based studies indicate a larger variety of bacterial residents may temporarily survive in this tick species (Benson et al., 2004; Narasimhan et al., 2014; Rynkiewicz et al., 2015; Van Treuren et al., 2015; Zolnik et al., 2016, 2018; Abraham et al., 2017; Cross et al., 2018; Landesman et al., 2019; Tokarz et al., 2019), but other reports suggest that many of the detectable bacteria from such studies may have been of environmental origin or introduced from contaminated reagents (Martin and Schmidtmann, 1998; Salter et al., 2014; Ross et al., 2018; Zolnik et al., 2018).

**Table 1:** Microbes detected in *I. scapularis*.

**Organism**	Identification methods^*^	Infection prevalence (developmental stage)^**^	**References**
**Bacteria, extracellular**			
*Acinetobacter* spp.	p	22% (L, N, A)	Narasimhan et al., 2014, 2017; Abraham et al., 2017; Thapa et al., 2019
*Borrelia burgdorferi*	i, d, v, p	30–72% (N, A)	Burgdorfer et al., 1982; Steiner et al., 2008; Aliota et al., 2014; Tokarz et al., 2019
*Borrelia mayonii*	i, v, p	0.65–2.9% (N, A)	Pritt et al., 2016; Cross et al., 2018; Johnson et al., 2018
*Borrelia miyamotoi*	p, d	1–5% (N, A)	Scoles et al., 2001; Tokarz et al., 2010, 2019; Cross et al., 2018; Johnson et al., 2018
*Corneybacterium* spp.	p	2% (L, N, A)	Narasimhan et al., 2014, 2017; Abraham et al., 2017; Thapa et al., 2019
Enterobacteriaceae	p	100% (N, A)	Van Treuren et al., 2015; Ross et al., 2018; Zolnik et al., 2018
*Enteroccocus* spp.	p	ND (L, N)	Narasimhan et al., 2014, 2017; Abraham et al., 2017
*Rhizobium* spp.	p	1% (L, N, A)	Zolnik et al., 2016; Thapa et al., 2019
*Rhodococcus* spp.	p	ND (N)	Rynkiewicz et al., 2015; Landesman et al., 2019
*Pseudomonas* spp.	p	1–100% (L, N, A)	Benson et al., 2004; Narasimhan et al., 2014, 2017; Zolnik et al., 2016, 2018; Abraham et al., 2017; Ross et al., 2018; Landesman et al., 2019; Thapa et al., 2019
Sphingomonadaceae	p	5% (L, N, A)	Benson et al., 2004; Zolnik et al., 2016, 2018; Abraham et al., 2017; Ross et al., 2018; Landesman et al., 2019; Thapa et al., 2019
*Staphylococcus* spp.	p	2% (L, N, A)	Narasimhan et al., 2014, 2017; Abraham et al., 2017; Zolnik et al., 2018; Thapa et al., 2019
*Streptococcus* spp.	p	ND (L, N)	Benson et al., 2004; Narasimhan et al., 2014
**Bacteria, intracellular**			
*Anaplasma phagocytophilum*	i, p	1.9–18% (N, A)	Adelson et al., 2004; Aliota et al., 2014; Cross et al., 2018; Tokarz et al., 2019
*Bartonella* spp.	p	13–90% (L, N)	Adelson et al., 2004; Rynkiewicz et al., 2015
*Ehrlichia* spp.	i, p	1.3–3.6% (N, A)	Aliota et al., 2014; Cross et al., 2018; Johnson et al., 2018
*Rickettsia buchneri*	i, v, p	46–100% (L, N, A)	Adelson et al., 2004; Narasimhan et al., 2014, 2017; Kurtti et al., 2015; Rynkiewicz et al., 2015; Zolnik et al., 2016; Cross et al., 2018; Tokarz et al., 2019
*Wolbachia* spp.	p	8–28% (A)	Zolnik et al., 2016; Cross et al., 2018
**Viruses**			
Blacklegged tick phleboviruses^+^	p	11–78% (A)	Tokarz et al., 2014a, 2018, 2019; Cross et al., 2018
Deer tick virus (Powassan virus lineage II)	i, p	0.4–4.7% (N, A)	Thomas et al., 1960; Telford et al., 1997; Dupuis et al., 2013; Aliota et al., 2014; Knox et al., 2017; Campagnolo et al., 2018; Johnson et al., 2018; Tokarz et al., 2019
*I. scapularis*-associated viruses^+^	p	0.5–4.5% (A)	Cross et al., 2018; Tokarz et al., 2018
Laurel Lake virus	p	ND (A)	Tokarz et al., 2018
Mononegavirus-like viruses^+^	p	2% (A)	Tokarz et al., 2014b; Cross et al., 2018
South Bay virus	p	20–52 (A) %	Tokarz et al., 2014b, 2018, 2019; Cross et al., 2018
Suffolk virus	p	10–17% (A)	Cross et al., 2018; Tokarz et al., 2018, 2019
**Fungi**			
*Beauveria* sp.	i	ND (N)	Ginsberg and LeBrun, 1996; Tuininga et al., 2009
*Cladosporium* sp.	i, p	ND (A)	Tuininga et al., 2009
*Colletotrichum* spp.	i, p	ND (A)	Tuininga et al., 2009
*Discostroma tricellulare*	i, p	ND (N)	Tuininga et al., 2009
*Hypocrea koningii*	i, p	ND (N)	Tuininga et al., 2009
*Metarhizium* sp.	i	ND (A)	Benoit et al., 2005; Tuininga et al., 2009
*Myrothecium verrucaria*	i, p	ND (N)	Tuininga et al., 2009
*Paecilomyces* spp.	i, p	ND (A)	Tuininga et al., 2009
*Penicillium* spp.	i	ND (A)	Tuininga et al., 2009; Greengarten et al., 2011
*Pestalotiopsis caudata*	i	ND (N)	Tuininga et al., 2009
*Phoma* sp.	i, p	ND (N)	Tuininga et al., 2009
*Verticillium fungicola*	i, p	ND (N)	Tuininga et al., 2009
*Verticillium lecanii*	i	4% (A)	Ginsberg and LeBrun, 1996; Zhioua et al., 1999
**Nematodes**			
*Acanthocheilonema* spp.	d, p	22–30% (N, A)	Namrata et al., 2014; Tokarz et al., 2019
*Onchocercidae* sp.	p	18% (A)	Cross et al., 2018
Unidentified microfilari	i, v	0.4% (A)	Beaver and Burgdorfer, 1984
**Protozoans**			
*Babesia microti Babesia odocoilei*	p p	3–20% (N, A) 1–15% (A)	Steiner et al., 2008; Tokarz et al., 2010, 2019; Aliota et al., 2014; Cross et al., 2018 Steiner et al., 2008; Hamer et al., 2014; Tokarz et al., 2019

* *i, isolation from I. scapularis; v, microscopic visualization; d, FISH, IFA or other method of indirect detection; p, PCR and sequence-based methods*.

** *Infection prevalence of individual ticks varied widely depending on the developmental stage of the tick (L = larva, N = nymph, A = adult) and the geographic location from which samples were collected. Confounding these factors was the method of calculating infection rates, some studies examined individual ticks while others pooled ticks and estimated individual infection rates*.

+ *Multiple viruses present within this grouping*.

*ND, not determined*.

The bacterial diversity of *I. scapularis* diminishes as the tick matures to the adult stage, with females having less diversity than males (Zolnik et al., 2016; Thapa et al., 2019). While many of these microbes are either stably maintained in *I. scapularis* and others are acquired from the environment and subsequently eliminated when the nutrients are depleted, it is likely that some microbes were erroneously attributed to the microbiome. Ross et al. concluded that *I. scapularis* lacks a stable bacterial microbiome, and those that are long-term residents are mostly intracellular (Ross et al., 2018). Microbiome studies using low biomass samples that are subjected to amplification and high-throughput sequencing often overestimate microbial diversity as a result of contaminating DNA present in reagents or from human skin (Salter et al., 2014), and this may provide an alternative explanation for the detection of some of the environmental bacteria. Further, sequencing errors and limited database sequences has led to the misidentification of at least one species in a microbiome study (Tijsse-Klasen et al., 2010). Likewise, Tokarz et al. disputed the identification of *Bartonella* as a component of the *I. scapularis* microbiome, attributing the mistake to a lack of specificity in primer design (Tokarz et al., 2019). Another factor that can inflate bacterial diversity estimates is the method of tick sterilization employed prior to sequencing, where ethanol-based surface sterilization of *Amblyomma cajennense* ticks correlated with reports of higher internal bacterial diversity than studies that used bleach-based methods, likely due to cuticular contaminants that were not removed by ethanol (Binetruy et al., 2019). Finally, detection of microbial DNA does not in and of itself prove that a specific species is a component of the microbiome. Organisms may be acquired with a bloodmeal but not survive, whereas their DNA may still be detectable. Due to the variety of issues that can arise in identifying microbiome members, it seems prudent that multiple techniques should be used to verify the members of any microbiome.

Most studies concur that *Rickettsia buchneri* is the predominant prokaryotic *I. scapularis* symbiont, and its relationship to the tick is significantly different from that of *B. burgdorferi*. An obligate intracellular bacteria, it appears to be able to establish itself in a variety of tissues including the salivary glands, midgut tissues, and ovaries (Noda et al., 1997; Kurtti et al., 2015; Zolnik et al., 2016; Al-Khafaji et al., 2020). *R. buchneri* is not known to infect vertebrate hosts but is an obligate symbiont of *I. scapularis* and transmitted transovarially from the mother to the progeny. All life stages of the tick can harbor *R. buchneri*, though it can reach exclusive levels in the adult female (Zolnik et al., 2016; Thapa et al., 2019). Its abundance in *I. scapularis* may relate to the ability of this prokaryote to provide folate (vitamin B9) to the tick, as animals are unable to synthesize this essential vitamin (Hunter et al., 2015). However, this does not appear to be an obligatory symbiotic relationship as *I. scapularis* ticks devoid of *R. buchneri* have been reported (Steiner et al., 2008; Tokarz et al., 2019).

A few human pathogenic bacteria are also well-documented symbionts of *I. scapularis* but with a wide variation in infection rates. *B. burgdorferi*, for example, is present in 30–64% of the ticks examined in areas endemic for Lyme borreliosis, while *Ehrlichia* species occurs in 1–3% of the ticks in the same geographic region (Table 1). Because of the potential overestimates of bacteria in the tick midgut due to the technical issues previously described (contaminated reagents and/or inefficient removal of DNA from the tick cuticle), further studies demonstrating interactions between *I. scapularis* and putative symbionts are required. Free-living, environmentally acquired bacteria may flourish within the fed midgut while nutrients from the bloodmeal are available. Although they may not persist transstadially, they may still impact the existing microbiota by competing for nutrients, altering the midgut environment (e.g., changing the pH), stimulating tick immune defenses, or by secreting toxic compounds (such as proteases or oxidative radicals).

Some bacterial symbionts may directly affect the fitness or behavior of their tick host. *A. phagocytophilum* was reported to induce an antifreeze glycoprotein of *I. scapularis*, thereby increasing the tick's cold tolerance (Neelakanta et al., 2010). Adult ticks infected with *B. burgdorferi* were less active and quested at lower heights compared to uninfected ticks, while infected nymphs had an increased phototaxis response and were more attracted to vertical surfaces than control nymphs (Lefcort and Durden, 1996). The effect on tick fitness of these altered behaviors remain unclear. As mentioned previously, *R. buchneri* may synthesize the essential vitamin B9, providing the tick with an essential nutrient that is scarce in the bloodmeal (Hunter et al., 2015). Although intriguing, such studies need further elaboration and are not likely to be the only interactions between the microbes and their tick host.

Although viruses associated with arthropods are common and collectively referred to as arboviruses (arthropod-borne viruses), few have been isolated and characterized from *I. scapularis*. Several PCR- and sequence-based studies have identified the *Bunyaviridae* South Bay virus and the Black-legged tick phlebovirus as putative symbionts of *I. scapularis* (Tokarz et al., 2014b, 2018; Sakamoto et al., 2016; Cross et al., 2018). Further research is needed to confirm the specific roles of these viruses in *I. scapularis*, but Tokarz and coworkers indicate a 3-fold greater viral diversity in *I. scapularis* relative to *Amblyomma americanum* (Tokarz et al., 2018). Deer tick virus (DTV), also known as Powassan virus lineage II, is a human pathogen and is the most well-characterized virus of *I. scapularis*. DTV, a flavivirus, was reported and named by Telford et al. in 1997 (Telford et al., 1997), but had previously been described in 1960 (Thomas et al., 1960). DTV is thought to display an interesting mechanism of tick acquisition in addition to being directly acquired from a viremic host. As demonstrated for other arboviruses, DTV may be acquired by cofeeding between uninfected and infected ticks (Jones et al., 1987; Labuda et al., 1993a,b). This, non-viremic mechanism occurs when multiple ticks are simultaneously feeding in proximity to each other, allowing the infected ticks to transmit the virus into the same blood pool that naïve ticks are also feeding from. Because DTV can be delivered into the host by an infected tick as early as 15 min after attachment (Ebel and Kramer, 2004), and the feeding period of *I. scapularis* takes days to over a week, the virus can amplify within the feeding lesion during this timeframe and be acquired by cofeeding naïve ticks. Transovarial transmission of DTV occurs at a relatively low rate (Costero and Grayson, 1996), but transstadial transmission (maintenance of the virus through the molt) is relatively inefficient, with only 20% of the newly molted ticks retaining the infection (Ebel and Kramer, 2004), indicating that DTV requires a vertebrate reservoir to be maintained.

Fungi and nematodes have been isolated from *I. scapularis* and these interactions are of some interest as entomopathogenic agents (Zhioua et al., 1995, 1997; Hill, 1998; Greengarten et al., 2011). Since the majority of the *I. scapularis* life cycle is spent on the ground in the same environment as many fungi and nematodes, it is not surprising that a variety of these species infect this tick. The invasive fungi produce proteases and chitinases that allow the hyphae to penetrate the exoskeleton and colonize the internal anatomy of the tick while nematodes may infect through the genital or anal openings. Some nematodes, though, are thought to be acquired by *I. scapularis* through the bloodmeal from infected animals (Namrata et al., 2014). Because nematodes and fungi may have their own microbiome, it is possible that some of the bacteria and viruses detected in the *I. scapularis* microbiome high-throughput sequencing studies might have originated from these parasites (Cross et al., 2018; Tokarz et al., 2019).

As shown in Table 1, the microbes that have adapted to infect *I. scapularis* are diverse and little is known about their ability to interact with each other. The bacterial symbionts and endosymbionts of *I. scapularis* (*Borrelia, Ehrlichia, Anaplasma*, and *Rickettsia*) lack most of the genes for mediating interbacterial interactions (Ross et al., 2018), suggesting they have evolved in an environment where these interfaces are unnecessary. However, potential microbe-microbe interactions may be indirect, where one organism modifies its environment (i.e., tick tissues) to the benefit or to the detriment of others.

*I. scapularis* can harbor multiple pathogens simultaneously (Table 2), raising the possibility of humans acquiring multiple microbial infections from a single tick bite. In a large serosurvey cohort study, patients with concurrent babesiosis and Lyme borreliosis had a greater number of symptoms and a longer duration of illness than patients with either infection alone (Kraiczy et al., 2001). For matters of human health, more research is needed to elucidate key aspects of polymicrobial infections and symbiont interactions within the tick.

**Table 2:** Coinfection of *I. scapularis* with human pathogens^*^.

**Pathogens**	**% of ticks coinfected**	**References**
**Dual infections**		
*B. burgdorferi – A. phagocytophilum*	1–26	Schwartz et al., 1997; Steiner et al., 2008; Tokarz et al., 2010, 2017, 2019; Aliota et al., 2014; Johnson et al., 2018; Sanchez-Vicente et al., 2019
*B. burgdorferi – Babesia microti*	1–22	Adelson et al., 2004; Steiner et al., 2008; Tokarz et al., 2010, 2017, 2019; Aliota et al., 2014; Johnson et al., 2018; Sanchez-Vicente et al., 2019
*B. burgdorferi – Bartonella spp*.	8	Adelson et al., 2004
*B. burgdorferi – B. miyamotoi*	1–3.5	Tokarz et al., 2017, 2019; Sanchez-Vicente et al., 2019
*B. burgdorferi –* DTV	0.4–2.5	Aliota et al., 2014; Tokarz et al., 2017, 2019
*A. phagocytophilum – B. microti*	1–2	Steiner et al., 2008; Tokarz et al., 2017
**Triple infections**		
*B. burgdorferi – A. phagocytophilum – B. microti*	1–8	Tokarz et al., 2010, 2017, 2019; Aliota et al., 2014; Sanchez-Vicente et al., 2019
*B. burgdorferi – B. microti – B. miyamotoi*	1–2	Tokarz et al., 2017

* *Only infections ≥ 1% are shown*.

## Evidence for Microbial Interactions

No direct mechanistic interactions have been experimentally defined among the microbiota of *I. scapularis*, but several studies have detected correlations between various organisms. The likelihood of *I. scapularis* being coinfected with both *B. microti* and *B. burgdorferi* was found to be higher than expected based on single infection rates (Dunn et al., 2014; Hersh et al., 2014; Edwards et al., 2019). This co-occurrence rate may potentially be explained by the existence of a host competent for both microbes, allowing acquisition of *B. microti* and *B. burgdorferi* in a single bloodmeal. In contrast, an interference effect was observed in ticks acquiring *A. phagocytophilum* and *B. burgdorferi* from infected *P. leucopus* (Levin and Fish, 2001). In these experiments, the primary agent inoculated into mice lowered tick acquisition rates of the second agent. However, in field-collected ticks from midwestern states, Hamer et al. found a higher than expected co-infection prevalence with *B. burgdorferi* and *A. phagocytophilum* (Hamer et al., 2014). This apparent discrepancy may relate to the former study examining the rate of both organisms being acquired simultaneously from an infected mammal while the latter report surveyed field-collected adults, which may have acquired the microbes in separate bloodmeals. Ross et al. reported that ticks highly colonized by species of the environmental bacteria *Bacillus, Pseudomonas*, and members of the Enterobacteriaceae had a lower frequency of infection with *B. burgdorferi* (Ross et al., 2018). In a multi-state study, Steiner and colleagues found that co-infections with *B. burgdorferi* and the endosymbiont *R. buchneri* were lower than predicted for male ticks based on individual infection prevalences (Steiner et al., 2008). Cross et al. collected individual ticks from Wisconsin and identified positive correlations (i.e., the presence of one microbe might predispose *I. scapularis* to infection by the other) between the presence of South Bay virus RNA levels and *B. burgdorferi*, South Bay virus and Blacklegged tick phlebovirus-1, and the *Onchocercidae* filarial worm and *Wolbachia* spp. (Cross et al., 2018). The correlation between *Wolbachia* and the filarial worm, though, may have been due to this bacterium being a natural component of the worm's fauna, and not as part of the tick microbiota (Cross et al., 2018; Tokarz et al., 2019). Analogously, *Wolbachia pipientis*, previously thought to be an endosymbiont of *I. ricinus*, actually derives from the endoparasitoid wasp *Ixodiphagus hookeri* which lays its eggs in the tick (Plantard et al., 2012). Cross et al. also detected a negative co-occurrence between Blacklegged tick phlebovirus-1 and−2, perhaps due to superinfection exclusion between closely related viruses (Cross et al., 2018). Tokarz et al. identified two positive correlations in ticks collected from New York and Connecticut: between *B. microti* and Blacklegged tick virus 1, and between Blacklegged tick phlebovirus and *B. burgdorferi* (Tokarz et al., 2019).

Experimental studies with *I. scapularis* produced interesting observations on the tick microbiome. Rearing *I. scapularis* in a sterile environment altered the tick microbiome compared to those reared in non-sterile conditions. These ticks with a perturbed microbiota were less efficiently colonized by *B. burgdorferi*, suggesting that the normal microbiome facilitates *B. burgdorferi* infection by an unknown mechanism (Narasimhan et al., 2014). Abraham et al. reported that *A. phagocytophilum* infection both weakens the peritrophic membrane of *I. scapularis* and inhibits bacterial biofilm formation, consequently altering the microbiome, but enhancing colonization of the tick by *A. phagocytophilum* (Abraham et al., 2017).

All of these lines of evidence suggest a rich interaction network among *I. scapularis* symbionts. In contrast to a wide variety of experimentally confirmed interfaces between microbes in insects, such interactions have not been demonstrated among the microbiota of *I. scapularis*. Partly this is due to the emphasis placed on vectors that have a greater impact on human health, such as mosquitos, and on agriculturally important pests. Additionally, development of biological tools has lagged behind those available in some insects, largely due to the relatively prolonged life span of ticks and their extended feeding period. Recent technical achievements, though, are producing rapid advances in our basic knowledge of tick physiology and its microbiome.

## New Dimensions for the Study of Tick Biology

Four recent developments provide the groundworks for rapidly advancing our understanding of *I. scapularis* biology and microbial interactions. These are: (1) application of RNA interference (RNAi) gene silencing methods for functional analysis of tick proteins; (2) the release of the annotated *I. scapularis* genome (Gulia-Nuss et al., 2016); (3) a contained, artificial feeding system for nymphal and adult *I. scapularis* (Oliver et al., 2016); and (4) *ex vivo* organ culture of midgut and salivary glands (Grabowski et al., 2017, 2019).

RNAi temporarily down-regulates gene expression through an incompletely understood pathway. Essentially, introduction of a double-stranded RNA template homologous to a target sequence will complex with endogenous proteins (including RNAseIII) that lead to degradation of the target RNA. As efficient targeted gene deletion techniques are not currently feasible in *I. scapularis*, RNAi gene silencing allows analysis of gene-product function in tick physiology and in microbial interactions. However, RNAi has limitations that need to be considered as the target transcript is only temporarily reduced and the existence of redundant proteins can mask the phenotype.

In conjunction with RNAi, the release of the annotated *I. scapularis* genome provides the sequence information necessary to design double-stranded RNA molecules for gene silencing experiments. The genome data also provide the complete sequence (introns/exons) of genes involved in pathogen acquisition, persistence and transmission by the tick, and allows identification of differentially regulated proteins in response to microbial infection using quantitative proteomic approaches (Gulia-Nuss et al., 2016). This assembly, although encompassing 1.8 Gb of sequence, does not close the 2.1 Gb genome, leaving about 14% of the genome yet to be completed.

Moving from the molecular to the organismal level, the development of an artificial feeding system opens up new avenues of research in tick biology. The lengthy feeding period of *I. scapularis* was problematic in obtaining fully engorged ticks from artificial feeding systems, as opposed to the long-standing success achieved with fast-feeding arthropods such as soft ticks, sandflies, fleas, and mosquitos (Hindle and Merriman, 1912; Adler and Theodor, 1927; Woke, 1937; Cerwonka and Castillo, 1958). Recently, a silicone-based membrane feeding system has been successfully adapted for *I. scapularis* to feed to repletion (Krober and Guerin, 2007; Oliver et al., 2016). After engorgement, nymphal ticks molted to adults and females mated and laid viable egg masses. Such membrane feeding systems have been useful for introducing specific pathogens into *I. scapularis* and to test different acaricides effectiveness on *I. ricinus* (Krober and Guerin, 2007; Oliver et al., 2016). Several recent studies have used these systems to elucidate the contribution of *B. burgdorferi* proteins in acquisition and transmission of mutant strains and to assess nutritional requirements for tick reproduction (Perner et al., 2016; Bernard et al., 2018; Hart et al., 2018; Koci et al., 2018). Larval ticks, though, did not successfully feed with this technique, likely due to the inability of the smaller mouthparts of larvae to penetrate through the thickness of the silicone membrane (Oliver et al., 2016). However, larvae can be infected with bacteria and viruses using the immersion technique first described by Policastro and Schwan (2003) (Mitzel et al., 2007; Policastro et al., 2011). The ability to acquire arboviruses has not been tested with this system, indicating a potential limitation in symbiont studies. Overall, the advantages of the artificial feeding system outweighs its disadvantages and opens new avenues to explore, including quantitation of pathogen transmission during feeding and effects on tick development of reducing the bacterial microbiota (dysbiosis) by addition of antibiotics in the bloodmeal. Symbionts can be reintroduced sequentially to dysbiosed ticks to study the individual contributions of each to the physiology of *I. scapularis* and to assess potential microbial interactions.

Symbiont—tick interactions can also be studied in specific tissues using the recently described *ex vivo* organ culture system described by Grabowski et al. (2017, 2019). Dissected midgut, salivary glands, and synganglion were maintained in a metabolically active state for 9–10 days. Powassan virus and Langat virus were able to infect and replicate in these artificially maintained organ cultures, indicating that this system can be used to study microbial infection and persistence in isolated tissues. These studies also demonstrated that RNAi could be used effectively in *ex vivo* organs to perturb virus replication. Although useful for the study of endosymbionts, it is not apparent how the *ex vivo* system might mimic the interactions between tick organs and extracellular bacteria with the influence of culture medium.

## A Model System for Studying Microbial Interactions Within *I. Scapularis*

Together, these resources open new avenues of investigation that were previously daunting to study. For example, DTV is designated as a BSL3-level agent in the U.S. and therefore feeding cohorts of infected ticks on animals becomes cumbersome and requires additional levels of containment. However, artificial membrane feeding chambers have been developed for safely containing and feeding infected ticks to repletion (Krober and Guerin, 2007; Oliver et al., 2016). DTV is the only native virus of *I. scapularis* that has been extensively studied *in vitro* and for which laboratory mouse and tick infectious models exist, making it an attractive candidate to study as a viral symbiont. *B. burgdorferi* represents an excellent model bacterium because it can be cultured *in vitro*, efficient molecular genetic tools exist to construct mutations, it is also efficiently transmitted in a laboratory mouse-tick infectious cycle, and it is a significant agent of disease in the United States. Although other bacteria listed in Table 1 would also be suitable, none possess all of the characteristics described above for *B. burgdorferi*.

Little evidence exists as to whether these two microbes interact within the tick. Because of the low rate of DTV circulating within wild *I. scapularis* populations, tick capture studies have not produced significant numbers of single or coinfections to compare prevalences. The question of whether *B. burgdorferi* impacts the ability of DTV or other microbial species to infect the same tick remains open. Attempts to address this question by examining European coinfection data is confounded by multiple factors: (1) different tick species transmit these microbial infections: *I. ricinus* in Europe and western Asia and *I. persulcatus* in Asia; (2) POWV is restricted to specific geographic regions in eastern Russia, whereas tick-borne encephalitis virus is more widely disseminated throughout Eurasia but is a distinct and separate flavivirus that has evolved in different tick species; and (3) multiple *Borrelia* species cause Lyme borreliosis in Eurasia. Therefore, *B. burgdorferi*—DTV—*I. scapularis* present a tantalizing combination for laboratory coinfection studies that permit characterization of symbiont interactions with each other and with the tick.

## Perspective and Conclusions

Ticks transmit the largest variety of pathogens of any arthropod vector, many of which are significant causes of medical and veterinary disease. In human health, dual infections transmitted by ticks may not only complicate accurate diagnosis and proper treatment but may potentially alter the symptoms (Krause et al., 1996; Djokic et al., 2019). The lack of licensed human vaccines in the U.S. for any tick-borne illness makes the study of *I. scapularis* and its microbiome imperative from a One Health perspective. Understanding the different components of the microbiome, their strategies for persistence within the tick, and their effects on each other and their host provide potential roadmaps in preventing tick-borne illnesses. However, this requires a more detailed knowledge of the microbial ecology existing within *I. scapularis*. First, we must distinguish between organisms adapted to using *I. scapularis* as a host or vector of transmission vs. those that only circumstantially and transiently interact with the tick. Focusing on the long-term passengers is more likely to be profitable because these microbes are adapted to and specific for *I. scapularis*, whereas generalists are widespread in the environment and may have pleiotropic effects on non-target organisms. Symbionts may be useful tools in themselves for disrupting the infectious cycle of pathogens, as they may be used as antagonistic symbionts, to engineer as paratransgenic microbes, or some may possess entomopathogenic properties. Further, the natural microbiota has identified vulnerabilities in *I. scapularis* physiology and evolved mechanisms to exploit these weaknesses to colonize and persist in the tick. These weaknesses in tick fitness may highlight pathways we can also exploit to design countermeasures (e.g., vaccines or therapeutics) against tick-borne diseases.

## Author Contributions

PS prepared the manuscript. MB critically reviewed the manuscript and provided thoughtful insights.

### Conflict of Interest

The authors declare that the research was conducted in the absence of any commercial or financial relationships that could be construed as a potential conflict of interest.
